# How to communicate and what to disclose to participants in a recall-by-genotype research approach: a multistep empirical study

**DOI:** 10.1007/s12687-024-00733-8

**Published:** 2024-09-26

**Authors:** Katharina Tschigg, Luca Consoli, Norbert Brüggemann, Andrew A. Hicks, Ciara Staunton, Deborah Mascalzoni, Roberta Biasiotto

**Affiliations:** 1 Department of Cellular, Computational, and Integrative Biology, University of Trento, Trento, Italy; 2 Institute for Biomedicine, Eurac Research, Bolzano, Italy; 3 Institute for Science in Society, Radboud University, Nijmegen, Netherlands; 4 Department of Neurology, University Hospital Schleswig-Holstein, Campus Lübeck, Lübeck, Germany; 5 Institute of Neurogenetics, University Hospital Schleswig-Holstein, Campus Lübeck, Lübeck, Germany; 6 School of Law, University of KwaZulu-Natal, Durban, South Africa; 7 Center for Research Ethics and Bioethics, Department of Public Health and Caring Sciences, Uppsala University, Uppsala, Sweden; 8 Department of Biomedical, Metabolic and Neural Sciences, University of Modena and Reggio Emilia, Modena, Italy

**Keywords:** Recall-by-genotype, Disclosure of genetic information, Communication, Research participants, Policy, Informed consent

## Abstract

Recall-by-genotype (RbG) is a bottom-up approach using existing genetic data to design follow-up stratified studies. Genetic information may be partially disclosed at invitation, thus raising ethical issues which call for defined best practices for disclosure and communication in RbG approaches. Within the context of the ProtectMove sub-project of the Cooperative Health Research in South Tyrol (CHRIS) study, we investigated research participant perspectives on RbG communication strategies (Step 1 and 4, questionnaire with a subsample of CHRIS participants with and without previous experience of RbG, respectively). Additionally, we explored researchers’ and study personnel’s experience with RbG (Step 2 and 3, focus group discussion). In step 1 (*N* = 95), participants were generally satisfied with the study process. Most (71.6%) wanted to know their carrier status for personal and collective benefit. Tailored disclosure strategies and transparent, effective, and well-thought-out communication approaches were advocated by study personnel (Step 2, *N* = 6) and researchers (Step 3, *N* = 7). Challenges in dealing with uncertainty, concerns caused by RbG invitations, and the possibility of misunderstanding were also raised. In step 4 (*N* = 369), participants valued being informed of study details at the first invitation stage, and generally felt comfortable towards RbG study invitations (58.5%) and to receiving genetic information after the study (58.5–81.6%). Comfort and perceived impact of disclosure of genetic information varied according to the type of variant being potentially disclosed. This study suggested designing communication strategies, based on clear and understandable explanations, sensitive to participant expectations and preferences, developing case-by-case solutions for disclosure.

**Supplementary Information:**

The online version contains supplementary material available at 10.1007/s12687-024-00733-8.

## Background

The availability of genetic, genomic, and other types of health data in biobanks has rapidly increased research opportunities with translational potential, while causing interest to proliferate in the ethical, legal, and social/societal implications (ELSI) of this type of research (Bledsoe 2012; Boyer et al. 2012; Cadigan et al. 2013). While the traditional approach to human genetics research relied on a phenotype-first approach, recent discoveries have been made using a ‘genotype-first’ approach (Mefford 2009; Wilczewski et al. 2023). In recent years, this advancement has led to targeted bottom-up approaches to participant selection, such as recall-by-genotype (RbG). The RbG or genotype-driven research (GDR) recruitment design (i.e., recruitment based on defined genetic characteristics of interest) has proven to be a powerful tool compared with traditional random sampling strategies. This is particularly so in cases where specific genetic characteristics are rare, and the phenotyping of large sample frames would be too costly (Atabaki-Pasdar et al. 2016; Franks and Timpson 2018; Minion et al. 2018; Corbin et al. 2018; Finer et al. 2020; Momozawa and Mizukami 2021). RbG studies reuse biobank material and data, thus decreasing possible public concerns about the underutilisation of biobank resources, which may undermine public trust (Cadigan et al. 2013, 2014; Klingler et al. 2022). However, RbG approaches raise issues concerning the involvement of participants (Minion et al. 2018; Corbin et al. 2018; Mascalzoni et al. 2021). Disclosure concerns have been explicitly highlighted because the disclosure of genetic information may be moved to the invitation phase (Beskow and Burke 2010; Beskow et al. 2012a, b). Thus, by inviting prospective study participants to an RbG study, they may incur partial or potential disclosure of their genetic information, possibly without being aware of its implications or without providing their personal decision to participate in these types of studies, especially if the possibility of participating in future RbG studies was not clarified nor asked during the original consenting process. The challenges of ethically managing recall and consent require careful assessment in various contexts, such as patient cohorts and healthy population cohorts, where participants’ expectations can differ significantly (Heinzel et al. 2022). While patients might anticipate and even welcome being re-contacted to gain deeper insights into their condition, the same assumption might not hold for healthy individuals.

Due to these ELSI challenges, policies for guiding the research are required. As part of this policy development, empirical research can contribute to identifying both issues and their solutions (Bergner et al. 2014; McGowan et al. 2018; Rutakumwa et al. 2019; Mwaka et al. 2021). While policies that govern participant data and novel study designs, such as RbG in biobanks, need more uniformity, there is no one-size-fits-all solution for such policies (Henderson et al. 2013; Lemke and Harris-Wai 2015). Stakeholder involvement and engagement have been used to provide ‘contextual evidence’ and inform specific practices and research (Krahn and Naglie 2008; Hoffman et al. 2010; O’Haire et al. 2011; Barry and Edgman-Levitan 2012; Carman et al. 2013; Fleurence et al. 2013; Lemke and Harris-Wai 2015). Further empirical research can facilitate the development of evidence-based guidelines informed by the experiences and opinions of stakeholders (Beskow and Burke 2010; Lemke and Harris-Wai 2015).

The present study investigated challenges of RbG communication strategies and participant preferences for disclosure approaches to inform the development of the Cooperative Health Research in South Tyrol (CHRIS) study’s policy regarding RbG research. The CHRIS study is an ongoing longitudinal, population-based study in Val Venosta/Vinschgau district, Italy (Pattaro et al. 2015). RbG studies have been conducted within the CHRIS study since 2018. We conducted a multistage empirical study that aimed to understand the perspectives of research participants, and also researchers and study personnel, on the RbG approach in the context of the CHRIS study, and thus contribute to the debate on ELSI issues in RbG studies, and to offer elements for policy building that may be generalisable to other contexts.

## Methods

### Aim

With this study, we investigated research participants’ preferences regarding communication and disclosure strategies in RbG approaches through a quantitative method (questionnaire). Additionally, we explored researchers’ and study personnel’s views on communication and disclosure challenges in RbG approaches with a qualitative method (focus group). Considering the perspectives and experiences of these groups, this study can shed light on crucial aspects of RbG communication strategies, inform CHRIS RbG disclosure policies and provide generalisable recommendations for best practices.

### Study context

#### RbG studies in the CHRIS study

In the past two decades, research into neurodegenerative diseases, such as genetic Parkinson’s disease (PD), has highlighted a range of causes, clinical manifestations, variations in populations, and increasing incidence and prevalence (Klein et al. 2007; Simon-Sanchez et al. 2009; van der Heide et al. 2021). Variants in the Parkin (*PRKN*) gene can cause genetic PD in homozygous individuals (Klein et al. 2007; Lesage et al. 2008; Simon-Sanchez et al. 2009). While evidence suggests that the heterozygous *PRKN* variant can increase the risk of some clinical symptoms related to PD or that it might be one of the potential genetic risk factors for PD, albeit at very low penetrance, the relationship between heterozygosity in this gene and the development of clinical or prodromal symptoms of Parkinsonism is not entirely clear, and may depend on other factors (Li et al. 2020; Castelo Rueda et al. 2021; Zhu et al. 2022). Further research is required to clarify the degree to which heterozygous carriers of a recessive variant (e.g., in *PRKN*) are at an increased risk of developing symptoms associated with PD, or whether protective factors can be identified that might prevent symptom onset.

Within the ProtectMove project (2024), the CHRIS study conducted an RbG study in August 2018 (CHRIS-PAREGEN, *PA*rkinson’s *RE*call by *GEN*otype, referred to hereafter as RbG1) (Prasuhn et al. 2021; Mascalzoni et al. 2021), followed by a subsequent RbG study in April 2022 (CHRIS-PAREGEN-2, from here referred as RbG2). Both were conducted at the CHRIS study centre in Silandro (Italy). These studies aimed to assess heterozygous carriers and non-carriers of *PRKN* variants without a PD diagnosis, using deep neurological phenotyping.

#### RbG2 study communication strategy

The RbG2 study design was similar to RbG1 (Mascalzoni et al. 2021). Accordingly, the communication strategy that was used in RbG2 was also similar to RbG1 (Biasiotto et al. 2023) and included the following:

**Table 1:** Framing of study design and objective in the invitation to participate in RbG2 study

	Framing
Study design	Recall-by-Genotype study. Physical recall study. Neurogenetic study. Standardized neurological examination.
Study objective	This study investigates: *PRKN* gene variants that may act as a weak risk factor for PD. Underlying biological mechanisms that contribute to or affect the development of PD or provide protection. Genotype-phenotype interaction mechanisms (reduced penetrance) with a focus on *PRKN* gene variants. Potential targets for therapeutic intervention. In-depth neurological examination.

- Participants were invited through a letter, and were provided with comprehensive and detailed information about the study, including its objectives, study design (Table 1), eligibility criteria, participant selection process, logistical details, examination procedures, associated risks and benefits, participant rights, informed consent process, voluntariness, data privacy, ethics approval, funding of the project.
- Participants were communicated the disease under investigation, PD, and the specific gene considered in the study, *PRKN*. They were not communicated the individual carrier status of variants under study.
- In the CHRIS study’s dynamic informed consent, participants can choose – and change over time – what types of genetic results they would like to receive in case Return of Research Results (RoRR) is applicable (Mascalzoni et al. 2022). The reasons for non-disclosure of the individual carrier status and the RoRR strategy (no return of research results) was explained: considering that carrying *PRKN* variants in the heterozygous state was not associated with any known clinical benefit (Mascalzoni et al. 2021; Biasiotto et al. 2023) and in line with established disclosure policy, the *PRKN* individual carrier status was not disclosed in either the initial invitation or after the study. To address previous findings (Staunton et al. 2021) on participants’ expectations or possible misconceptions regarding the return of unsolicited results, it was crucial to provide clear and transparent information on this implication of participation. No compensation was foreseen.
- For further information and clarification, dedicated CHRIS study centre contacts (phone number and email) and the option to speak with a neurologist were made available.

### Study design

This study was designed as a multistage empirical investigation, including quantitative and qualitative approaches. We described the whole process through “steps”, which included a series of empirical investigations, each with different interlocutors and different aims, in a temporal succession (from 1 to 4). Building upon previous findings (Mascalzoni et al. 2021; Staunton et al. 2021; Tschigg et al. 2022; Biasiotto et al. 2023), we designed the following steps, which are described in Table 2:

1. Survey with CHRIS participants who participated in the RbG2 study.
2. Focus group discussion (FGD) with CHRIS study personnel.
3. FGD with researchers with experience in RbG approaches.
4. Cross-sectional online survey submitted to a sample of the CHRIS cohort. Results obtained in Step 2 and 3 contributed to informing the design of the survey in Step 4.

**Table 2:** Study design

	Methods	Participants	What was investigated	Aim
Step 1	Questionnaire with close and open-ended questions and scoring question	CHRIS participants with experience of RbG study approach consisting of participation in an RbG study on heterozygous *PRKN* variants and PD	Satisfaction with the RbG study design Experience with PD Willingness to receive information about carrier status Self-assessment of non-carrier likelihood Essential aspects of participation in genetic research	To understand research participant perspectives on RbG communication strategy, namely: - participant experience with the study process - participant views on information disclosure related to the specific type of variant and disease under study
Step 2	Focus group discussion	CHRIS study personnel, which are in direct contact with participants throughout the informed consent process and the overall study	Disclosure and framing, peculiarities of RbG studies and ELSI challenges	To discuss RbG-specific ELSI challenges as perceived by study personnel in the process of performing the RbG study, namely: - to understand their experience on the practical aspects of the RbG approach
Step 3	Focus group discussion	Researchers with experience in RbG studies	Study design (recruitment, sampling and implicit or explicit disclosure) Future studies and differing degree of (potential or partial) disclosure of results for participants	To discuss RbG-specific ELSI challenges as perceived by researchers in the process of performing the RbG study, namely: - to understand researchers’ perspectives on essential aspects, difficulties, and concerns related to the design and execution of RbG studies
Step 4	Questionnaire with close and open-ended questions	CHRIS participants without previous experience of RbG studies	Feelings (comfort) about RbG invitations and disclosure strategies Importance and timing of potentially receiving information Preferences for disclosure strategies concerning different types of genetic variants Response to an hypothetical RbG approach with no disclosure of study details	To understand research participant perspectives on RbG communication strategies, namely: - to understand research participant preferences regarding possible RbG disclosure policies

In the Discussion, we integrated quantitative and qualitative findings through a narrative approach (Fetters et al. 2013) and discussed the findings in relation to developing recommendations for best practices and policy adjustment on RbG studies.

#### Step 1: Survey with RbG2 study participants

##### Survey RbG2 data collection

In April and May 2022, a survey was administered to participants of the CHRIS study who had also taken part in the RbG2 study. The questionnaire, available in German and Italian (in Vinschgau/Val Venosta district where the study is conducted, German is the mother tongue for most (Pattaro et al. 2015; Mascalzoni et al. 2022), is shown in English in Additional file 1). The survey was completed online at the study centre using the LimeSurvey platform, with study personnel available to assist participants and address any questions.

##### Survey RbG2 data analysis

Quantitative data were analysed using descriptive and statistical methods in Microsoft Excel (Microsoft Excel Spreadsheet Software, Microsoft 365, 2023) and SPSS (SPSS Software, 2023), while open-ended questions were analysed through qualitative content analysis (Creswell 2014) in German and Italian. The results were then translated and discussed with all the authors.

#### Steps 2 and 3: Focus group discussions

We conducted two FGDs in June 2022. The overall aim was to discuss RbG-specific ELSI challenges as perceived by study personnel and researchers in the process of performing the RbG study. We followed relevant guidelines for conducting, analysing and reporting findings (Wong 2008).

##### Step 2. Focus group discussion with study personnel: recruitment and data collection

An FGD was conducted in person with CHRIS study personnel at the CHRIS study centre in the hospital of Silandro. The inclusion criterion was RbG study experience in the CHRIS study.

We developed informative materials to clarify the terminology related to RbG approaches, and four hypothetical versions of an RbG study invitation letter, which differed in the level of details provided. The discussion guide and the translated exemplary invitation letters are shown in Additional file 2. The FGD was conducted in German (the mother tongue of the FGD participants and the moderator), moderated by one author (KT), and observed by another author (DM). The FGD lasted approximately 90 min.

##### Step 3. Focus group discussion with researchers: recruitment and data collection

An FGD was conducted online with researchers with experience in RbG approaches within the CHRIS study and the ProtectMove study. For the sampling strategy, we used a combination of purposive and snowball sampling (Singh and Moodley 2021). Participants were recruited by email, and the FGD, conducted in English, explored research-related aspects of RbG approaches. The inclusion criterion was experience with the design or ethical assessment of RbG studies. The discussion guide was developed from discussions among colleagues and is shown in the Additional file 2. The FGD, led by one author (KT) and observed by another (DM), lasted approximately 90 min.

##### Focus group discussion data analysis

Audio recordings of the discussions were transcribed for analysis. KT analysed transcripts using qualitative content analysis (Hsieh and Shannon 2005; Creswell 2014), and the results were discussed with the authors. Descriptive summaries of the main findings were produced and selected quotes (originally in English or translated into English) to illustrate the key considerations were included. The analysis was performed in the respective language of the FGD.

#### Step 4: Survey with CHRIS baseline participants

We developed a cross-sectional survey that included extensive information material through an iterative review process with research group leaders who had conducted research within the CHRIS study and its coordinator. The design of the questionnaire and the explanation material were informed by the issues identified in the FGDs (Step 2 and 3). For some questions, the answer choices were informed by the results obtained in Steps 1 and previous research (Biasiotto et al. 2023). Furthermore, we used ‘think aloud’ methods (Kernebeck et al. 2022) to test the content of the information material, survey questions, and answer choices with the study personnel and discussions with the CHRIS Study coordinator regarding comprehension and adequate language for the participants.

##### Survey CHRIS data collection

Data collection occurred in April 2023. A sample of 1,712 CHRIS baseline participants was invited via email to answer an online questionnaire hosted by LimeSurvey (“LimeSurvey — Free Online Survey Tool,” 2023). The sampling strategy was designed to recall participants from the CHRIS baseline study who had also taken part in the subsequent ongoing follow-up (CHRIS 2) and who had not been previously approached for participation in recent sub-studies to avoid possible participation overburdening. The survey was available in German and Italian according to each respondent’s language preferences. Respondents had two weeks to complete the survey.

The questionnaire (provided in Additional file 3) comprised closed- and open-ended questions.

##### Survey CHRIS data analysis

Only fully answered questionnaire responses were considered for the analysis. Demographic data were used as descriptor of the sample. We used Excel (Microsoft Excel Spreadsheet Software, Microsoft 365, 2023), and SPSS (SPSS Software, 2023) for the descriptive statistics of the quantitative data in the form of Likert-scale questions and single- or multiple-choice questions. We used qualitative content analysis for the open-ended questions that were coded and categorised (Hsieh and Shannon 2005).

## Results

### Step 1. Survey with RbG2 participants

#### Characteristics of respondents

All of the RbG2 participants (*N* = 95) completed the survey after completing their examinations in the study centre in Silandro. There were 53 male and 42 female participants aged 51–81 years.

#### Satisfaction concerning study process and information materials

The vast majority of respondents (93 respondents, 97.9%) felt like their questions had been answered during the study process, and a very small minority felt like not all of their questions had been answered (2 respondents, 2.1%). Similarly, most respondents were satisfied with the obtained clarifications (Table 3). Most participants had positive feelings about the invitation process and the rationale behind the study. They viewed the information material and the informed consent process positively. The study team, including personnel and medical doctors performing the examination, were positively perceived by the most.

**Table 3:** Respondents’ satisfaction with the RbG study components

	Very negative *n*/*N* (%)	Somewhat negative *n*/*N* (%)	Neutral *n*/*N* (%)	Somewhat positive *n*/*N* (%)	Very positive *n*/*N* (%)	Blank *n*/*N* (%)
Invitation	0/95 (0.0)	0/95 (0.0)	4/95 (4.2)	4/95 (4.2)	85/95 (89.5)	2/95 (2.1)
Study rationale	0/95 (0.0)	0/95 (0.0)	4/95 (4.2)	6/95 (6.3)	80/95 (84.2)	5/95 (5.3)
Information material	0/95 (0.0)	0/95 (0.0)	7/95 (7.4)	10/95 (10.5)	70/95 (73.7)	8/95 (8.4)
Informed consent	0/95 (0.0)	0/95 (0.0)	7/95 (7.4)	5/95 (5.3)	75/95 (78.9)	8/95 (8.4)
Clarification of questions	0/95 (0.0)	0/95 (0.0)	2/95 (2.1)	7/95 (7.4)	79/95 (83.2)	7/95 (7.4)
Study personnel	0/95 (0.0)	0/95 (0.0)	0/95 (0.0)	3/95 (3.2)	87/95 (91.6)	5/95 (5.3)
Medical doctors	0/95 (0.0)	0/95 (0.0)	0/95 (0.0)	2/95 (2.1)	88/95 (92.6)	5/95 (5.3)

#### Key considerations when participating in genetic research

In an open-ended question, 36 respondents (37.9%) clarified perceived priorities and most crucial aspects when participating in genetic research, including personal benefits, such as receiving results and findings and gaining insights into one’s health (e.g., staying informed about test results, discovering abnormalities, learning about risks/prevention, acquiring relevant health information, and learning about incisive disease factors and control); and collective benefits, such as helping and contributing to science, research study and development of targeted therapies, with an adequate level of privacy and discretion.

#### Willingness to know the individual carrier status and reasons

Most respondents (68 respondents, 71.6%) were willing to know their carrier status, while a minority (20 respondents, 21.1%) did not want to know the information on the carrier status; and very few (7 respondents, 7.4%) expressed that they did not care about knowing their carrier status.

The primary reason to know carrier status was to prepare for the possible onset of symptoms and hereditary reasons (Table 4).

**Table 4:** Motivation for wanting or not wanting to know one’s individual carrier status based on expressed relatability to the provided reason

Motivation	Yes *n*/*N* (%)	No *n*/*N* (%)
Want to know because of heredity	28/95 (29.5)	67/95 (70.5)
Want to know to prepare for the possible onset of symptoms	34/95 (35.8)	61/95 (64.2)
Maybe want to know, but haven’t thought enough about it	11/95 (11.6)	84/95 (88.4)
Do not have to know, it is acceptable not to disclose the individual carrier status	11/95 (11.6)	84/95 (88.4)
Do not want to know to avoid worrying	9/95 (9.5)	86/95 (90.5)
Do not need to know	13/95 (13.7)	82/95 (86.3)
Do not want to answer	7/95 (7.4)	88/95 (92.6)

Additionally, four respondents chose “Other”, and in the open field, they expressed interest in knowing the individual carrier status, while one would not want to know.

#### Self-assessment of the likelihood of carrying the variant under study and experience with PD

The respondent’s perceptions of their likelihood not to carry the studied variants exhibited a wide range, from 0 (unlikely) to 100 (very likely). The most frequently chosen likelihood is 50 (28 respondents, 29.5%), the median of all responses is 50, and the average is 40.6.

Of the 95 respondents, 24 (25.3%) reported having experience with PD in their families; the rest reported no experience (*N* = 63, 66.3%) or being unsure (*N* = 8, 8.4%). We did not find a significant effect of a familial history of PD or the decision of wanting to know one’s individual carrier status.

### Step 2. Focus group discussion with the study personnel

A total of six CHRIS study personnel participated in the FGD. They worked as nurses, assisted participants in the informed consent process and collected data and biological samples. The study personnel highlighted concerns about potential misunderstandings by participants and difficulty explaining the reason for the invitation without causing concerns. For example, they viewed the RbG study design based on matching carriers and non-carriers as confusing and potentially causing concerns related to a possible perceived increased risk of health issues (Table 5, Q1).

**Table 5:** Selected quotes from the focus group discussions with study personnel (step 2) and researchers (step 3)

Focus group discussion	Quote (Qn)
Step 2. Study personnel	Q1: The possibility of a disease is much more concrete. If you are selected by genotype to participate in a study, then the risk or the possibility is actually 50/50. (P1)
	Q2: The follow-up studies are actually something nice that people feel like they are not once “studied” but more often studied and therefore become part of the research. (P2)
	Q3: Yes, that direct communication in the field [in the study centre] is what makes the study so interesting because they put in the effort no matter if they get something [results or compensation] or not; they [the participants] would already come out a bit smarter about the situation. (P3)
Step 3. Researchers	Q4: And still, if you have kind of 50/50 cases and/or controls and carriers, you still have a much higher probability of being a carrier than if you were just randomly drawn from the population. So, it (the RbG study invitation) still discloses information. (P1)
	Q5: I think that it’s really difficult to generalise. That is the type of disclosure because there are some diseases or some arguments that we just imagine; epilepsy or dementia are more delicate than others. And then you have to really define the communication also to the population that you are considering and these things. So, if you have to, just suggest some things, it depends really on the case from the study that you are studying; there is not a general rule in my case. (P2)
	Q6: And of course, there are certain risks and benefits involved both in disclosure and concealment, but wouldn’t you say that it should be the participant’s choice whether they want to have disclosure or concealment? Of course, we have to kind of explain it to them well enough. (P3)

To mitigate this concern, the study personnel emphasised the need to offer clarifications and address these concerns in the recall phase. They proposed strategies for ensuring the clear comprehension of information and striking a balance between providing information and preventing information overload such as employing user-friendly electronic tools for communication (e.g., apps and websites). When discussing hypothetical invitations with different degrees of information and disclosure, it was unanimously agreed that the foremost critical information is the associated disease. They found that the best version explained the study design, rationale, and associated disease but did not disclose the specific genetic variant or individual carrier status. In their view, it is crucial not to overwhelm participants at the beginning with too many details; instead, one should use a layered approach as regard timing and modality, for example, providing further explanations in a face-to-face setting. The concept of sensitivity, as the ability to be considerate of other people’s feelings (expressed by the German word “Feingefühl”), was suggested for tailoring the disclosure and communication approach to the study context. The growth of the biobank, leading to more studies and more engagement, was perceived to deepen the relationship between the study personnel, doctors, researchers, and participants and to develop their role as partners (Table 5, Q2).

The study personnel perceived that in-person explanations were valued by participants as a means of increasing awareness and genetic literacy concerning research in general and the specific disease under study in each sub-study (Table 5, Q3).

### Step 3. Focus group discussion with researchers

In Step 3, seven researchers with expertise in different fields and roles in research (i.e., epidemiologist, study coordinator, scientist, and ethics board member) participated.

For researchers, participants’ perspectives and preferences were crucial to the design and implementation of RbG studies: considering participants’ views would contribute to promote respectful and transparent engagement without objectification or misunderstandings about the implications of participation. Communicating complex scientific concepts in an understandable manner (e.g., transparent communication without overwhelming scientific jargon) was perceived as a challenge because of a lack of specific training in the field and the possible risk of unwanted medicalisation of healthy individuals. Sampling and recall strategies were deemed critical, highlighting challenges in identifying controls and managing participants’ consent for follow-up studies. Debates arose regarding the disclosure of individuals’ carrier status and the study objectives, with uncertainty complicating effective communication. It was noted that an RbG study participant had a higher probability of being a carrier than if they were randomly drawn from the population (Table 5, Q4).

The researchers agreed that clear communication is crucial but challenging when uncertainty exists about the utility or consequences of a particular genetic variant. They agreed with the difficulty of identifying generalised rules for disclosure so that case-by-case solutions would be required (Table 5, Q5).

Regarding how to approach disclosure policies, some researchers suggested diluting or enlarging subsamples to ease burdens. Others emphasised the importance of participants’ choice to have disclosure or concealment (Table 5, Q6).

The tailoring of approaches was focused on the timing of disclosure (i.e., the possibility of disclosing the carrier status after participation) and adhering to participants’ preferences. Tailoring disclosure policies according to the specific case was perceived as necessary to avoid skewing the results. This could occur due to explicit or implicit knowledge of an individual’s carrier status affecting their perception and performance in the RbG study.

### Step 4. Large-scale survey with CHRIS participants

#### Characteristics of CHRIS survey respondents and feedback

We obtained a response rate of 24.3%, from 416 respondents. After removal of incomplete surveys, the final data set comprised 369 respondents for the analysis. Age and gender characteristics of respondents are shown in Table 6.

**Table 6:** Socio-demographic characteristics of survey respondents

**Age**	
Median age	53 years
Age range	29–90 years
Group 29–39 years old, *N* (%)	73 (19.8%)
Group 40–49 years old, *N* (%)	81 (22.0%)
Group 50–59 years old, *N* (%)	102 (27.6%)
Group 60–69 years old, *N* (%)	77 (20.9%)
Group 70–79 years old, *N* (%)	31 (8.4%)
Group over 80 years old, *N* (%)	5 (1.4%)
**Gender**	
Female, *N* (%)	213 (57.7)
Male, *N* (%)	156 (42.3)

Forty-two respondents (11.4%) provided general feedback, on the following:

1. Satisfaction and trust: (a) appreciation for the opportunity to participate in the CHRIS study; (b) positive experiences and comfort during the study; and (c) expression of trust in the CHRIS study and team.
2. Suggestions for improvement: (a) simplified language (replacement of complex medical terms with simple and understandable language); (b) desire to receive findings and summary of aggregated results.
3. Interest in research and further participation: willingness to participate in future studies in general and on specific diseases.

#### Reception of RbG invitation and reasons

A total of 216 respondents (58.5%) expressed that they would feel comfortable in receiving the invitation to participate in the RbG study; 90 respondents (24.4%) were indifferent; and 56 respondents (15.2%) indicated that they were uncertain about how they felt regarding the invitation, and one would rather not answer (0.3%). Only two participants (0.5%) stated that they were not comfortable with the invitation, and four participants (1.1%) chose “other”.

The primary reasons for feeling comfortable were a positive experience with their previous participation in the CHRIS study, and a desire to contribute to scientific knowledge and develop better therapies (Table 7). Most respondents had no particular reason to feel uncomfortable participating in a hypothetical RbG study. Accordingly, most of those who chose “other” expressed that they did not feel uncomfortable nor worried, or had no reason to feel uncomfortable (67.9% of those who chose “other”). Some respondents expressed worries about negative findings for their health or of their family (Table 7).

**Table 7:** Reasons for feeling comfortable or uncomfortable about participating

Reason for feeling comfortable	*n*/*N* (%)	Reason for feeling uncomfortable	*n*/*N* (%)
Good experience with CHRIS	129/369 (35.0)	Worries about negative health/genetic findings for oneself/family	90/369 (24.4)
Felt as a duty	3/369 (0.8)	Concern about not understanding the study	23/369 (6.2)
Sense of solidarity, benefit of society and future generations	27/369 (7.3)	Unwillingness to invest time and effort in participating	4/369 (1.1)
Contributing to scientific knowledge and the development of therapies	108/369 (29.3)	Disinclination towards participating in smaller studies	0/369 (0.0)
Gaining knowledge about one’s health, e.g., blood or urine tests	53/369 (14.4)	No particular reason	207/369 (56.1)
Gaining knowledge about genetic risk factors for oneself or own family	39/369 (10.6)	Would rather not answer	17/369 (4.6)
No particular reason	7/369 (1.9)	Other	28/369 (7.6)
Would rather not answer	1/369 (0.3)		
Other	2/369 (0.5)		

#### Preferences for type of information to be disclosed at invitation

Table 8 shows that the majority of participants definitely would like to know the disease under investigation; which genetic variant is being analysed; whether clinical results, such as blood test results, are returned; and whether information on genetic risk factors is offered. A large proportion of respondents were indifferent towards the reason for invitation of selected participants and the availability of medical doctors to ask further clarifications.

**Table 8:** Participant preferences for information in the invitation letter before the RbG study

		Preferences, *n*/*N* (%)			
		I would definitely like to know	Indifferent	I would rather not know	I prefer not to answer
Type of information	Disease	326/369 (88.3)	34/369 (9.2)	7/369 (1.9)	2/369 (0.5)
	Genetic variant	286/369 (77.5)	74/369 (20.1)	7/369 (1.9)	2/369 (0.5)
	Reason for selective invitation	180/369 (48.8)	168/369 (45.5)	13/369 (3.5)	8/369 (2.2)
	Return of clinical results	336/369 (91.1)	31/369 (8.4)	1/369 (0.3)	1/369 (0.3)
	Availability of genetic information	321/369 (87.0)	41/369 (11.1)	6/369 (1.6)	1/369 (0.3)
	Availability of medical doctors	255/369 (69.1)	113/369 (30.6)	0/369 (0.0)	1/369 (0.3)

#### Reception of invitation without specific details

Most respondents expressed negative feelings (very negative: 74 respondents, 20.1%; rather negative: 163 respondents, 44.2%) about receiving an invitation without detailed information about the genetic variant and the disease under investigation. Some expressed indifference (58 respondents, 15.7%) and uncertainty (I don’t know: 30 respondents, 8.1%). On the other hand, a minority had positive feelings (rather positive: 30 respondents, 8.1%; very positive: 8 respondents, 2.2%) about this approach. One respondent (0.3%) preferred not to answer, and 5 respondents (1.5%) chose “other”.

Some respondents (*N* = 49) explained their choice in the open-field text, from which we identified the following considerations:

- Desire for information: Some respondents expressed a clear desire to be informed about any potential diseases or health conditions (‘I support genetic research, but I want to know what it is for and about which disease’), while others expressed indifference or a lack of interest in being informed about diseases.
- Concerns about lack of information: Participants expressed a range of emotional responses, including worry, anxiety, concern, fear, and increased uncertainty, to not being informed about the genetic variant and associated disease (e.g., ‘I would immediately worry because I would think that there is a risky situation that I am not yet informed about’).
- Acceptability of non-disclosure: Some respondents mentioned the role of review bodies and ethical and legal aspects when deciding whether to provide information about diseases (e.g., ‘I would welcome being informed, but if the committee cannot inform due to ethical and legal reasons, then I accept that’).
- Trust in the research and scientific process and desire to contribute: Respondents expressed a willingness to contribute to research (e.g., ‘I am willing to participate if it is beneficial for research’) and trust in the researchers (e.g., ‘I trust the scientists conducting the study’).
- Importance of transparency and communication: Respondents emphasised the importance of transparency in the study, especially regarding using their data and avoiding disinformation or feeling objectified (e.g., ‘I want to know what is being researched with my data’).
- Expectation and reciprocity: Participants expressed the desire to contribute to research but also expected some benefit or feedback regarding the results or findings of the study. Several respondents expect a level of reciprocity for their participation, indicating that if they contribute to research, they should receive valuable information in return.

#### Disclosure of individual genetic information after an RbG study: feelings, concerns, and impact

Most respondents felt comfortable potentially receiving information about their genetic data (disclosure of individual carrier status) for disease-causing/pathogenic genetic variants, probably pathogenic genetic variants, benign genetic variants, and protective genetic variants (Table 9). On the other hand, for genetic variants with uncertain clinical significance, there was a decrease of answers expressing comfort and an increase of answers reporting indifference, feeling uncomfortable, and uncertainty (Table 9).

**Table 9:** Feeling comfort in receiving individual genetic information (disclosure of carrier status) for various types of genetic variants after participating in an RbG study

		Feeling, *n*/*N* (%)				
		Comfortable	Indifferent	Not comfortable	Do not know	Would rather not answer
Type of individual genetic information	Disease-causing/pathogenic genetic variants	292/369 (79.1)	31/369 (8.4)	8/369 (2.2)	32/369 (8.7)	6/369 (1.6)
	Likely pathogenic genetic variants	271/369 (73.4)	40/369 (10.8)	10/369 (2.7)	44/369 (11.9)	4/369 (1.1)
	Benign genetic variants	297/369 (80.5)	37/369 (10.0)	4/369 (1.1)	27/369 (7.3)	4/369 (1.1)
	Protective genetic variants	301/369 (81.6)	36/369 (9.8)	3/369 (0.8)	25/369 (6.8)	4/369 (1.1)
	Genetic variants of uncertain significance/ uncertain clinical significance	216/369 (58.5)	73/369 (19.8)	22/369 (6.0)	52/369 (14.1)	6/369 (1.6)

Some respondents (*N* = 20) justified their choices, and we identified the following general considerations:

1. Importance of being informed: A shared belief existed that receiving information about one’s genetic characteristics is essential. The participants expressed that being informed would allow for preventive measures, early detection, or appropriate actions.
2. The desire for detailed descriptions: Participants emphasised the need for clear and comprehensive descriptions of the genetic variants and their implications. They valued receiving informative and understandable information, especially regarding potential disease risks or preventive measures.
3. Preference for consultation with healthcare professionals: Some respondents preferred to consult with healthcare professionals to understand their genetic variants’ significance better. They believed professional guidance would be essential for accurately interpreting the information and making informed decisions about their health.

We found differences in the level of interest, perceived relevance to personal health, emotional impact, and understanding according to the type of genetic variant under investigation (Table 10).

**Table 10:** Overview of the responses regarding different types of genetic variants

	Disease-causing/pathogenic variants	Probable pathogenic variants	Benign variants	Protective variants	Genetic variants of uncertain significance
Level of interest	High	High	Lower (compared with disease-causing and probable pathogenic variants)	Lower (compared with disease-causing and probable pathogenic variants)	Mixed responses (including disinterest, concerns about uncertainty, and desire for clear results)
Perceived relevance to personal health	Highly relevant	Relevant	Less relevant	Less relevant	Relevance was uncertain, with varying degrees of importance attributed to these variants
Emotional impact and responses	Worry, anxiety, and the desire for additional cautiousness	Worry and the desire for additional precautions	Provision of reassurance and reduction of worry	Provision of reassurance and reduction of worry	Mixed responses (expressing worry or a desire for clearer information)
Perceived level of understanding of implications	Well-understood and acknowledged to have significant implications	Some uncertainty regarding their exact implications; professional guidance should be sought for interpretation and understanding	Understood as indicating a lower risk or no significant health impact	Potentially helpful in guiding lifestyle choices or behaviours	Professional guidance should be sought to interpret and understand the significance

Disease-causing and probable pathogenic variants evoked a higher level of interest, while benign and protective variants were considered less relevant but offered reassurance or potential benefits. Variants of uncertain significance elicited mixed responses, including desire for more evident results and professional guidance, disinterest and concerns about uncertainty.

## Discussion

This study was part of a broader multistage investigation within CHRIS and the ProtectMove project, which included a feasibility assessment and stakeholder engagement (Mascalzoni et al. 2021), and an empirical study with CHRIS participants who participated in an RbG sub-study on Parkinson’s disease (RbG1) (Biasiotto et al. 2023). By surveying participants of a second RbG study focused on the assessment of heterozygous carriers of *PRKN* variants and non-carriers from the CHRIS cohort (RbG2) and a cross-sectional survey with a sample of CHRIS participants without previous experience of RbG approaches, we gained insight into participant perspectives and disclosure preferences. These were enriched by the communication and disclosure trajectory considerations that emerged during two focus groups, with researchers and CHRIS study personnel. This study incorporated the obtained empirical results into considerations for a balance between researchers’ needs and fundamental legal and ethical guidelines, and recommendations for best practices on RbG policy development.

### Expectations on communication

In the large-scale survey (Step 4), most respondents expressed negative feelings about the possibility of receiving an invitation without detailed information. They would want to be informed upfront, principally about the disease being investigated, the variants and their potential consequences or lack thereof, the return of clinical results, and the availability of genetic risk factors information, thus showing a keen interest in the health implications of the study. Participant expectations for information aligned with expectations of transparency, reciprocity, and trust were also found in other studies (Minion et al. 2018; Biasiotto et al. 2023).

To further contextualize the meaning and the health implications of carrying a certain variant, it would be important to inform and educate research participants on the concept of penetrance. For example, in RbG studies with 50/50 design (50% carriers of a specific variant of interest, and 50% non-carriers), 50% of participants might expect to carry the variant under study, but this could mean very little chance of “getting the disease” if the penetrance is low. This should be clarified for the participants in such studies to avoid confusion and misleading assumptions and contribute to reducing possible negative emotional responses upon receiving the invitation to participate in studies about low penetrant variants. Clarification of complex genetic concepts such as penetrance seems to be in line with the wish expressed by participants in this study (Step 4) for clear, comprehensive, and understandable information on the variants under study and their implications. Using suitable media and strategies for tailored communication would be key for delivering such concepts (Rothwell et al. 2023). Considering that the degree of penetrance may affect participant desire for disclosure of genetic risk information, as shown in Viberg-Johannson et al. 2019, educating about penetrance and providing specific information on penetrance in each RbG study acquires an even more important role in the communication with participants.

### Disclosure of carrier status

As observed in a previous RbG study (Nurm et al. 2022), we identified a positive attitude toward the disclosure of carrier status in both surveys. In Step 4, participants reported feeling generally comfortable about disclosing individual genetic results after participating in an RbG study. However, different genetic variants with differing impacts on health were deemed to be received with different interests and need for interpretation of implications. When considering the specific variants under investigation in RbG2 (*PRKN* variants in heterozygosity, which could slightly increase the risk of some attenuated PD clinical symptoms but with very low penetrance), most participants were willing to know the individual carrier status and they perceived this awareness to be beneficial due to potential passing on to their children and potential onset of symptoms considerations (Step 1). In a previous empirical study embedded in a similar RbG (RbG1), the nondisclosure of the *PRKN* variant carrier status was generally deemed acceptable, and participants were okay with genetic research results not being returned to them as they perceived their value within the research context only (Biasiotto et al. 2023). The primary considerations for the acceptability of non-disclosure of *PRKN* variant carrier status included the perceived low relevance for the personal health (Biasiotto et al. 2023). Even though the RbG1 and RbG2 studies shared the main focus, the participant sample differed regarding age (older in RbG2), the embedded empirical studies used different approaches (mixed method in Biasiotto et al. 2023, survey in this study) and different investigations (e.g., acceptability of non-disclosure was not explored thoroughly in this study), thus possibly reflecting the different results of these studies. The willingness of participants to know their carrier status, primarily driven by concerns for potential clinical symptoms and hereditary considerations, emphasizes the importance of providing relevant health information, if in line with participants’ wishes.

A recent study in Italy reported that most patients expressed a desire to be informed about any gene variant, including those that may not be clinically actionable (Godino et al. 2021), while another study reported that participants were confident in using the individual results from whole genome sequencing (WGS) to prevent future disease, and believed in the value of even uninterpretable information, among other factors that were motivated by wanting to contribute to their, or their relatives, health positively (Facio et al. 2013). As observed in other studies, collecting expectations regarding the communication of results from WGS is crucial for avoiding disappointment (Suckiel et al. 2021). According to stakeholder perspectives, providing research participants with their results demonstrated respect for their autonomy and interests (Tindana et al. 2020; Singh and Moodley 2021).

Researchers determine the process of recall, the decision to offer individual genetic results, and how to disclose them, often in consultation with institutional or external ethical review boards, where many contextual factors are considered. Decisions on disclosure and RoRR can be facilitated if researchers are aware of participants’ expectations through empirical research or ongoing engagement and tailor the plans and policies accordingly. Participants might want to know if they have a genetic risk of a particular disease; however, a worry exists that such information, with or without clinical utility, could lead to undue anxiety, stigma, or discrimination (Beskow et al. 2004, 2012b; McGuire and Lupski 2010; Bledsoe et al. 2013; Beskow 2017). Participants may also not want to know at all (right not to know). There are diverging views and discussions on how to define categories (from clinical to personal utility), which contextual factors should be prioritised in the assessment, what should be disclosed, and whether ethical obligation takes precedence over preferences (Beskow and Burke 2010; Cassa et al. 2012; Matsui et al. 2021). We found that case-by-case solutions would be required from the researchers’ perspective, depending on the utility of the information, the nature of the disease, the study design, and participants’ preferences and choices on the right to know and not to know. Results on the impact and interest of different genetic variants disclosure (obtained in Step 4) may contribute to clarify the priority criteria according to participants’ perspectives. As with good practical research decisions, ethical judgement requires contextual knowledge (Hammersley 2009), and context matters when deciding on whether and how to offer individual genetic research results (Beskow and Smolek 2009; Beskow and Burke 2010; Bledsoe 2012).

A key focal point of the ongoing discourse surrounding RoRR is how RbG research methodologies impact the level of disclosure obligations. Before disclosing any genetic variant in an RbG study, evaluating whether it meets the disclosure criteria according to established guidelines is crucial (Matsui et al. 2021). In numerous cases, the usual criteria or thresholds for actionability of the carrier status information may not be satisfied (Beskow et al. 2010, 2012a; Budin-Ljosne et al. 2013). A coordinated best practice has yet to be defined regarding how and when to disclose or communicate the carrier status of genetic variants with unknown actionability or utility to participants.

Various recommendations for evaluating the responsibility for communication to a participant include assessing various factors, such as the strength of association or availability of treatment options, have been proposed (Cassa et al. 2012). Still, if one or more of these factors are unknown or missing, the responsibility to disclose is uncertain (Cassa et al. 2012). A previous study (Staunton et al. 2021) in the CHRIS context, investigating opinions of general practitioners and the genetic counselling service in Bolzano, emphasised the need for clinical validation before returning research results and case by case assessment.

### How to tailor communication

#### Tailoring invitation and information material to participant preferences for disclosure

Challenges related to designing the invitation letter and study-related materials particularly in terms of transparency and managing expectations, were identified (Leitsalu et al. 2021, 2022). In RbG studies design, legal obligations, such as disclosing the study’s nature and purpose, can be perceived differently depending on how the information is presented. Different ways of framing the information regarding an RbG study may result in different reception and perception by the invited participants. RbG2 participants in Step 1 reported satisfaction with the information provided before (see Table 1 for the type of information provided in the letter) and during the RbG2 study. In a sort of feedback loop, participation and engagement may result in increased health and scientific literacy (as suggested in previous studies (Beskow et al. 2010, 2012a; Budin-Ljosne et al. 2013; Leitsalu et al. 2022), which may benefit the understanding of future studies, as implied by the study personnel in the FGD (Step 3).

In the FGDs, concern for framing was also presented as particularly challenging because of difficulties in providing participants with clear and understandable explanations of the study design, eligibility criteria, and utility and consequences of carrier status awareness (especially in cases of uncertainty). Therefore, it would be important to clarify the contribution of specific RbGs in resolving the uncertainty on the impact of carrying a certain variant: one of the purposes of specific RbG studies is indeed to provide clearer evidence as to consequences (or none) of carrying the variants under study. Other identified difficulties lay in avoiding overburdening participants, preventing invitation fatigue, communicating about genetic variants without medicalising healthy individuals, and implications of genetic information at the familial level. Echoing findings from earlier studies (O’Haire et al. 2011; Deverka et al. 2012), we suggest that employing consistent terminology throughout interactions with participants would be beneficial for clear and effective communication over different disclosure strategies and implications.

To tailor information material to different preferences, it is crucial to consider participants’ attitudes towards the disclosure of their carrier status. On the one hand, transparency is crucial for ensuring full understanding, making informed decisions about participation, and the realistic expectations about RoRR and the study outcome (Leitsalu et al. 2022). On the other hand, transparency regarding the study’s purpose and participation eligibility should not be at the expense of the individual’s (or relative’s) right not to know genetic information (Beskow et al. 2011, 2012a; Beskow 2017). While the CHRIS study participants in this study with and without previous experience with RbG approaches seemed interested in receiving information on the individual carrier status after participation regarding different types of genetic variants, overall, there were various motivations for this, that encompassed the perceived relevance for health, emotional impact, and other implications. Accordingly, communication strategies for RbG studies could be based on previously defined choices on disclosure which accommodate different preferences for disclosure of different types of individual genetic information in the recall and for timing of layers of information and may be changed over time through a dynamic consent model. This would allow participants to participate in sustainable long-term dialogue with researchers about the type of information they wish to be disclosed, while at the same time meeting requirements for transparency. Additionally, in order to improve comprehension, such a dynamic consent model may be designed to provide supplementary materials using a multimedia approach, to explain technical terms and concepts in plain language, regarding specific aspects of the RbG approach, such as the potential implications of the eligibility and disclosure possibilities for each specific RbG study, and the degrees of specificity regarding study details to enable tailored communication. Nevertheless, such a tool would only be helpful if individuals can, and want to, access and engage with it (Teare et al. 2021).

#### Personal contact and balanced involvement

A large part of the satisfaction reported by participants in the large-scale survey (Step 4) was related to familiarity, comfort, and the professional conduct of the CHRIS study personnel. Previous studies have recommended the possibility of participants being recontacted by someone who is ‘trusted’, ‘familiar’, or ‘known’ to them to enhance participant satisfaction and comfort (Beskow et al. 2012a; Budin-Ljosne et al. 2013). Providing a welcoming environment, respectful and empathetic attitudes, and personalised interactions, becomes crucial in contributing to a positive research experience and engagement. In parallel, a coordinated and balanced communication strategy (with defined frequency and type of communication that participants can expect) based on less intrusive methods, such as online public communication, should be developed to ensure that participants do not become overburdened through their involvement. While such engagement methods are less intrusive in their informing and consulting functions, estimating their impact and uptake is challenging (Lemke and Harris-Wai 2015).

## Strengths and limitations of the study

We adopted a multistep approach, including various research methods and stages. This allowed a more nuanced understanding of the diverse considerations associated with disclosure in RbG studies, from specific (RbG2 study) to generalized situations (possible future RbGs with different types of variants), ensuring that the policy development process was informed by those involved in RbG studies, primarily by research participants, and enriched by considerations of researchers and study personnel. Possible future investigations aiming to develop policy may consider using other quantitative methods to detect preferences and extend these investigations to other contexts quantitatively. To gather further evidence, we recommend including more and different ELSI experts (such as research ethics committee members, policymakers, and regulators), and research participants from different settings. This broader engagement will contribute to a more comprehensive understanding of the governance and oversight of biobanks in RbG research.

## Conclusion

This study consisted of a multistage empirical investigation that explored research participants’ perspectives on RbG studies, with the contribution of study personnel and researchers’ views, and aimed to inform the development of effective communication and disclosure strategies and policies.

Considering the empirical studies on RbG study design conducted so far, divergences exist regarding the ELSI of RbG studies, with respect to, for example, the recall strategies and return of individual genetic research results (Beskow and Burke 2010; Beskow et al. 2012a). These divergences reflect heterogeneity in viewpoints and approaches to disclosure, communication strategies, and study-specific policies related to RbG, highlighting the necessity for tailored approaches. Our study underscored the importance of crafting communication strategies that are sensitive to individual preferences, emphasising the need for clear and understandable explanations of study details. Transparency, reciprocity, and trust emerged as critical pillars that participants highly value in the communication and dissemination of study information.

## Electronic supplementary material

Below is the link to the electronic supplementary material.

Supplementary Material 1

Supplementary Material 2

Supplementary Material 3

## Data Availability

The data generated and analysed in the present study are CHRIS study data. CHRIS study data can be requested for research purposes by submitting a dedicated request to the CHRIS Access Committee by contacting access.request.biomedicine@eurac.edu.

## Abbreviations

CHRIS: Cooperative Health Research in South Tyrol
ELSI: Ethical, legal, and social/societal implications
FGD: Focus group discussion
GDR: Genotype driven research/recall/recruitment
RbG: Recall by genotype
RoRR: Return of research results
PD: Parkinson’s disease
PRKN: Parkin gene
