# Supplementary material for: How to communicate and what to disclose to participants in a recall-by-genotype research approach: a multistep empirical study
Source: J Community Genet. 2024 Sep 26;15(6):615–30. doi: 10.1007/s12687-024-00733-8 (PMC11645387; doi:10.1007/s12687-024-00733-8)
Supplement: Supplementary file 1 — Supplementary Material 1 [file 12687_2024_733_MOESM1_ESM.docx]

**Questionnaire for Step 1: a survey with a sample of CHRIS participants who participated in a specific RbG study.**

The original questionnaire was made available for respondents in German and Italian. Below is reported a shortened schematic version translated into English which shows the questions, the answer options, and the format of the questionnaire.

*These were not analysed in this study, as they were used for internal purposes only.

|  | **Question** | **Answer options** | **Format** |
| --- | --- | --- | --- |
| **A** | Were all your questions answered today? | Yes  Not all  No | Single choice |
| **A1** | If a question has not been clarified, please feel free to list it here. | Open field |  |
| **A2** | How would you rate today's study?   - Invitation - Study rationale - Information material - Informed consent process - Methods of the study* - Clarification of questions - Study personnel - Medical doctors - Study length* | Very negative  Somewhat negative  Neutral  Somewhat positive  Very positive | Single choice |
| **A3** | If you can think of any other important factors, please feel free to include them here: | Open field |  |
| **B** | Do you have someone with Parkinson's in your family? | Yes  No  I do not know  Prefer not to answer | Single choice |
| **C** | Would you like to know which group you belong to (carriers or non-carriers)? | Yes  No  I do not care  Prefer not to answer | Single choice |
| **D** | Which of the followings corresponds to your opinion?   - I want to know my individual carrier status because of heredity and children. - I want to know my individual carrier status so that one can prepare for it. - Maybe I want to know, but I haven't thought enough about it. - I don't have to know, it is acceptable not to disclose the individual carrier status. - I don't want to know to avoid worrying. - I do not need to know. - I do not want to answer. - Other (please specify). | Yes  No | Single choice per item |
| **D1** | You are also welcome to give your own explanation as to why you would like to know the information about the individual carrier status or not. | Open field |  |
| **E** | How likely do you think it is that you belong to the control group that does not carry the gene variants under study? | 0-100  (0= unlikely, 100 very likely) | Scoring |
| **F** | When I participate in a genetic study, the most important thing for me is that: | Open field |  |
| **G** | What is the maximum duration of the study?* | 1 hour  2 hours  3 hours  4 hours  5 hours | Single choice |
| **H** | Do you want to tell us anything else about today's study? | Open field |  |
