# Supplementary material for: How to communicate and what to disclose to participants in a recall-by-genotype research approach: a multistep empirical study
Source: J Community Genet. 2024 Sep 26;15(6):615–30. doi: 10.1007/s12687-024-00733-8 (PMC11645387; doi:10.1007/s12687-024-00733-8)
Supplement: Supplementary file 2 — Supplementary Material 2 [file 12687_2024_733_MOESM2_ESM.docx]

**Question guides of the focus group discussions with the study personnel and researchers**

**Step 2: Focus Group Discussion with the study personnel**
A ppt presentation was used to guide the discussion and elicit discussion. Discussion proceeded through the following steps:

1. Introduction

- Warming up: Encourage participants to share their thoughts and ideas through photo elicitation. For example, show a picture while asking a question.

1. Discussion on the Recall-by-genotype (RbG) approach:

First, terminology and study design were explained:

- Explanation of terminology:
  1. Genotype: The individual genomic/DNA information of study participants, which is collected through genetic screenings that examine the entire DNA of the participant.
  2. Phenotype: The manifestation of characteristics or diseases collected through examinations and surveys.
  3. Environment: The individual exposure to various external factors is collected through surveys and other data.
- Explanation of RbG study design in the context of the CHRIS cohort through visual aids elicitation material. Four cases of hypothetical invitation letters representing different framings and levels of disclosure were shown.

**Disclosure cases**

The four invitation letters vary in their approach to disclosure and communication. While all letters share common elements, they differ in their level of granularity and details about study-specific information, such as the study design, the purpose of the study, the disease under investigation and the disclosure strategy of the eligibility criterion. The first case provides the least specific information, while the second case narrows down the focus to Parkinson's disease. The third case places a strong emphasis on the method of neurological phenotyping. The fourth case provides the most detailed information, including explicit carrier status disclosure. Below, the summary of the four invitations:

**1^st^ Case**: inviting participants to a genetic study in the CHRIS cohort with no further specifications on disease or genetic variant under study.

**2^nd^ Case**: inviting participants to a genetic study in the CHRIS cohort with a specific focus on Parkinson's disease with no further specifications on the genetic variant under study.

**3^rd^ Case**: inviting participants to a genetic study in the CHRIS cohort with a specific focus on Parkinson's disease, emphasising the method (neurological phenotyping) and rationale**.**

**4^th^ case**: inviting participants to a genetic study in the CHRIS cohort with a specific focus on Parkinson's disease, communicating the method, i.e., neurological phenotyping, and explicitly disclosing the individual carrier status information.

Then, the discussion covered the following:

- Perceived differences between RbG studies and other follow-up studies.
- Ethical, legal, and social issues (ELSI) with examples.

1. Discussion on RbG-specific ELSI challenges:

First, an overview of ELSI challenges of the RbG approach with respective examples was presented through elicitation material covering the following:

- Violation of the person's right not to know.
- Participants may receive results of screenings with uncertain clinical significance.
- Participants may not understand why they are invited to participate in the RbG study.
- Emotional distress for participants and their families, including anxiety and burdens.
- No results as motivation or compensation.
- Duration and complexity of study examinations.
- Complexity of study and issues that may be difficult for participants to understand.
- Potential confrontation of family members with genetic information if the implications of the study's specific variant are relevant to them.

Then, the discussion covered the following:

- General ELSI challenges related to RbG studies.
- Specific RbG-ELSI challenges according to their experience in assisting RbG studies conducted so far.
- Hypothesizing possible scenarios for future RbG studies.
- Final remarks.

**Step 3: Focus Group Discussion with Researchers**

The focus group discussion was structured as follows:

1. Introduction
2. General discussion
3. Case discussion.
4. Introduction:

- Welcome participants
- Short presentation of the research team and the significance of the contribution expected from participants for the project
- Short presentation of the researchers and their work and role.

1. General discussion: A ppt presentation was used to guide the discussion and elicit views on the following:

- Warming up: choose a cartoon and relate it to themselves.
- Factors that are most relevant when designing a RbG study, using a link provided to write their answers.
- Reasonings behind sampling and recruitment strategy choices.
- Implicit or explicit disclosure of genetic information through the study invitation to an RbG study.
- Ethical, legal, and social issues (ELSI) challenges related to RbG studies.

1. Case discussion: Participants were challenged with a scenario (based on literature) of heterogeneous research participant preference for disclosure and motivators for participation. The discussion was led by the following questions:
   1. How do you account for the preferences of participants if they are split?
   2. What rules should be used to decide on disclosing the carrier status or not?
   3. Participants might feel like a guinea pig if they are not informed about why they are re-invited: how to avoid such sentiments in participants?
   4. How should researchers invite participants and family members while preventing the “is there something wrong with me/our family?" concerns of participants?
   5. Can you hypothesise possible scenarios for future RbG studies?
   6. Any final remarks? Have we missed anything?
