# Supplementary material for: How to communicate and what to disclose to participants in a recall-by-genotype research approach: a multistep empirical study
Source: J Community Genet. 2024 Sep 26;15(6):615–30. doi: 10.1007/s12687-024-00733-8 (PMC11645387; doi:10.1007/s12687-024-00733-8)
Supplement: Supplementary file 3 — Supplementary Material 3 [file 12687_2024_733_MOESM3_ESM.docx]

**Questionnaire for Step 4, survey with a sample of CHRIS participants without previous experience of RbG studies.**

The original questionnaire was made available for respondents in German and Italian. Below is reported a shortened schematic version translated into English which shows the questions, the answer options, and the format of the questionnaire.

| **Nr** | **Theme, Question** | **Answer options** | **Format** |
| --- | --- | --- | --- |
| A | **A brief general explanation of recall-by-genotype studies** | | |
| A1 | Suppose you are invited by the CHRIS study team to participate in a Recall-by-Genotype study.  Question: How do you feel about being invited to participate in a recall-by-genotype study based on your genotype information? | - Comfortable; I would welcome the invitation - Indifferent; I have no particular feeling about the invitation - Not comfortable - I don't know - I would rather not answer - Other | Single choice. If Other is selected, specify in the open field (optional). |
| A2 | Question: What is the main reason you would feel comfortable participating? Please select 1 reason that applies or enter another. | - I had a good experience with CHRIS - I felt it was my duty - I felt a sense of solidarity for the benefit of society. future generations - I want to contribute to scientific knowledge and the development of better therapies - To gain knowledge about my health such as blood / urine tests in CHRIS Baseline - To gain knowledge about genetic risk factors for myself or my family - No particular reason - I would rather not answer - Other | Single choice. If Other is selected, specify in the open field (optional). |
| A3 | Question: What is the main reason why you would feel uncomfortable participating? Please select 1 reason that applies or indicate another. | - I am concerned that something negative might be found out about my or my family’s health or genetic risk factors - I am concerned about not understanding well what the study is about - I do not want to bother or take the time to come to the CHRIS study center - I don’t like participating in smaller studies that don’t include the whole cohort - No special reason - I would rather not answer - Other | Single choice. If Other is selected, specify in the open field (optional). |
| B | **Content of the invitation letter to the Recall by Genotype study** | | |
| B1 | Suppose the CHRIS study team invites you to participate in a recall-by-genotype study.  Question: What information would you like to receive in the invitation letter BEFORE the Recall-by-Genotype study, and how important is the specific information to you?  Please select the importance of the different information:   - Which disease is being investigated - Which genetic variant is being studied - The reason why some CHRIS participants are invited and others are not - Whether clinical results are returned (e.g. blood results) - Whether information about genetic risk factors is offered - Whether a doctor is available to ask questions | - I would definitely like to know - Indifferent - I would rather not know - I prefer not to answer | Single choice per each information. |
| **C** | **The possible disclosure of carrier status in relation to different genetic variants and associated diseases** | | |
| C1 | Question: What do you think about, AFTER the Recall-by-Genotype study, possibly getting information about your genetic data and the different types of genetic variants and diseases?   - Disease-causing/pathogenic genetic variants - Probably pathogenic genetic variants - Benign genetic variants - Protective genetic variants - Genetic variants of uncertain significance/ uncertain clinical significance | - Comfortable; I would welcome the invitation - Indifferent; I have no particular feeling about the invitation - Not comfortable - I do not know - I would rather not respond | Single choice per each type of genetic variant |
| C2 | Explanation of choice:   - Disease-causing/pathogenic genetic variants - Probably pathogenic genetic variants - Benign genetic variants - Protective genetic variants - Genetic variants of uncertain significance/ uncertain clinical significance | Open field | Possibility to explain in open field specifically for each type of variant. |
| **D** | **No disclosure of study details (which genetic variant and which disease)** | | |
| D1 | Question: How would you feel if the invitation told you that you were invited for "further genetic research" and did not inform you about the genetic variant and the disease considered in the study? | - Very negative - Rather negative - Indifferent - Rather positive - Very positive - I don't know - I would rather not answer - Other | Single choice. If Other is selected, specify in the open field (optional). |
| D2 | Would you like to explain your answer? | Open field | Open field |
| **E** | **General and demographics** |  |  |
| E1 | If there is anything else you would like to share, please feel free to do so here: | Open field | Open field |
| E2 | Which gender do you identify yourself with?  Please select the answer that applies. | Female  Male  Other / non-binary |  |
| E3 | When were you born?  Please enter your date of birth. |  |  |
